# Facilitator-guided vs self-guided debriefing in immersive virtual reality paediatric emergency training: a randomised pilot study on learning outcomes and feasibility

**DOI:** 10.1007/s00431-026-06898-3

**Published:** 2026-04-02

**Authors:** Amalie Middelboe Sohlin, Ida Madeline Hoffmann, Anja Poulsen, Line Klingen Gjærde, Todd P. Chang, Joy Yeonjoo Lee, Gritt Overbeck, Stine Lund, Jette Led Sørensen, Jesper Kjærgaard

**Affiliations:** 1 Department of Paediatrics and Adolescent Medicine, Copenhagen University Hospital – Rigshospitalet, Juliane Marie Vej 8, 2100, Copenhagen, Denmark; 2 Mary Elizabeth’s Hospital and Juliane Marie Centre, Copenhagen University Hospital – Rigshospitalet, Copenhagen, Denmark; 3 Children’s Hospital Los Angeles, Keck School of Medicine of University of Southern California, Los Angeles, USA; 4 Faculty of Governance and Global Affairs, The Hague, Leiden University, Leiden, Netherlands; 5 Department of Public Health, Faculty of Health and Medical Sciences, University of Copenhagen, Copenhagen, Denmark; 6 Department of Paediatrics, Copenhagen University Hospital North Zealand, Hillerød, Denmark; 7 Department of Clinical Medicine, Faculty of Health and Medical Sciences, University of Copenhagen, Copenhagen, Denmark

**Keywords:** Simulation, Virtual reality, Immersive technologies, Paediatric emergency medicine, Debriefing, Feasibility

## Abstract

This study evaluated the feasibility and effectiveness of immersive virtual reality (VR)-based paediatric emergency training, comparing facilitator-guided with self-guided debriefing methods. In a single-blinded, randomised controlled pilot trial, 24 healthcare professionals (12 interprofessional dyads) were randomised to VR-based paediatric emergency training followed by either facilitator- or self-guided debriefing. Both groups received a standardised preparatory lecture and skills workshop. We collected data on the feasibility of the interventions and additional trial procedures. For pre- and post-performance assessment, all dyads were video-recorded whilst managing a mannequin-based paediatric emergency. Two independent, blinded raters assessed the dyads’ teamwork, ABCDE adherence, and time to critical actions based on the videos. We also collected data on participants’ cognitive load and perceived effectiveness of the two debriefing methods. The study demonstrated the feasibility of interprofessional VR-based paediatric emergency training with both facilitator- and self-guided debriefing in a clinical setting. Twenty-two healthcare professionals completed the intervention, and 20 completed follow-up. VR was acceptable and usable, though 46% of participants reported some cybersickness. Additional trial procedures were feasible but required substantial logistical coordination. VR training significantly improved teamwork (facilitator-guided mean 1.2, confidence interval (CI) 0.5 to 1.9, *p* = 0.005; self-guided mean 1.4, CI 0.8 to 2.0, *p* < 0.001) and ABCDE adherence (facilitator-guided mean 0.5, CI 0.1 to 1.0, *p* = 0.02; self-guided mean 0.5, CI 0.1 to 0.9, *p* = 0.01), with similar outcomes between the debriefing methods. However, participants perceived the facilitator-guided debriefing to be more effective (mean difference 1.4, CI 0.1 to 2.7, *p* = 0.04).

*Conclusion*: Immersive VR-based paediatric emergency training with facilitator-guided and self-guided debriefing was feasible in a pilot context and yielded comparable performance outcomes. A larger, adequately powered trial is warranted to formally evaluate non-inferiority between debriefing methods.

**Table:** 

**What is Known:**	
• *Simulation-based paediatric emergency training improves clinical outcomes, but widespread implementation is limited by logistical challenges and high costs.*	
• *Immersive virtual reality (VR) may help overcome these barriers, yet evidence on its feasibility and effectiveness in this context is limited.*	
**What is New:**	
• *VR-based paediatric emergency training is feasible and effectively improves interprofessional performance.*	
• *Self-guided and facilitator-guided debriefing yielded comparable outcomes, highlighting the potential for scalable use.*

**Supplementary information:**

The online version contains supplementary material available at 10.1007/s00431-026-06898-3.

## Introduction

Rapid recognition and management of critically ill children are vital to improving quality of care and survival in paediatric emergencies [1, 2]. Yet the low frequency of such events limits healthcare professionals’ opportunities to gain and maintain competence through clinical experience alone. Simulation-based paediatric emergency training has been shown to significantly enhance clinical skills and improve patient outcomes [3–5]. However, regular training is needed to maintain these skills [6–8] and simulation-based training can be resource-intensive and costly [9].

Immersive virtual reality (VR) is an emerging modality for simulation-based paediatric emergency training, offering learners a highly engaging and interactive training environment [10–16]. VR can be programmed to adapt dynamically to learners’ actions, allowing learners to train without the need of an in-person facilitator [17]. This flexibility offers potential advantages in terms of scalability, cost-effectiveness, and ease of access—particularly for settings where regular facilitator-led training may not be feasible.

A core component of simulation-based training is the debriefing. Debriefing can be described as a guided reflection aiming to explore and learn from the simulation-based experience [18, 19] and is associated with large positive effects on learning outcomes [18–24]. Facilitator-guided debriefing is widely considered the gold standard [25], but self-guided debriefing—where learners use structured aids such as reflection guides—has potential advantages, such as increasing ownership and motivating self-regulated learning [26, 27]. Yet learners with low self-regulated learning skills may feel insecure evaluating their own performance progress, perceive the self-guided debriefing as less effective, and experience a high cognitive load [28, 29].

Whilst facilitator-guided and self-guided debriefing have been compared in other simulation modalities, such as mannequin-based and screen-based simulation [30, 31], little is known about the feasibility and effectiveness of self-guided debriefing in VR. If VR is to fully deliver on its promise of independent, scalable training, it is crucial to examine the feasibility of this complex intervention, and to determine whether self-guided debriefing can be as effective as the facilitator-guided approach.

The aim of this study was to address the following research questions (RQ):

- RQ1 (feasibility). Are the interventions acceptable and usable (RQ1a), and does this pilot study provide evidence that a future, larger randomised controlled trial is feasible (RQ1b)?
- RQ2 (effect on performance). Is self-guided debriefing comparable to facilitator-guided debriefing in improving paediatric emergency performance (RQ2a) and is immersive VR effective for improving performance with both debriefing methods (RQ2b)?
- RQ3 (perceived effectiveness and workload). Are there differences in perceived effectiveness and cognitive load between self-guided and facilitator-guided debriefing?

## Methodology

### Participants and research design

#### Study design

A randomised, controlled, single-blinded pilot study with a parallel group, pretest–posttest design, following the Consolidated Standards of Reporting Trials Statement extension for Pilot and Feasibility Trials [32].

A total of 24 healthcare professionals were included, organised into 12 interprofessional doctor-nurse dyads, and randomised to immersive VR simulation with either facilitator-guided or self-guided debriefing. For pre- and post-performance assessment, at baseline and at 1 month follow-up, each dyad was video-recorded managing a mannequin-based paediatric emergency. Two independent raters blinded to group allocation assessed the dyads’ paediatric emergency performance based on the videos.

#### Population

The study was conducted at the Department of Paediatrics and Adolescent Medicine, Copenhagen University Hospital, Rigshospitalet. Eligible participants were postgraduate trainee doctors and postgraduate nurses at four paediatric units (Paediatric Emergency Department, Paediatric Semi-Intensive Care Unit, Paediatric Bone Marrow Transplant Unit, and Department of Neonatology). Exclusion criteria were medical doctors who had completed specialist training and nurses with more than 10 years of postgraduate clinical experience. Participants were contacted by email and invited to volunteer. Informed consent and permission to record and store all video data for the research project were obtained from all participants upon enrolment.

#### Sample size calculation and randomisation

This pilot study was based on a convenience sample of 24 participants, determined by feasibility and participant availability. As no formal sample size calculation was performed, the study was exploratory in nature and designed to assess feasibility and generate preliminary data to inform a future larger randomised trial.

We formed interprofessional doctor-nurse dyads based on schedule availability and randomised the dyads to the intervention or control group at a 1:1 allocation ratio. The randomisation sequence was generated using Sealed Envelope’s online randomisation service [33].

### Materials

#### Development of immersive VR scenarios and core game mechanics

**Fig. 1:**
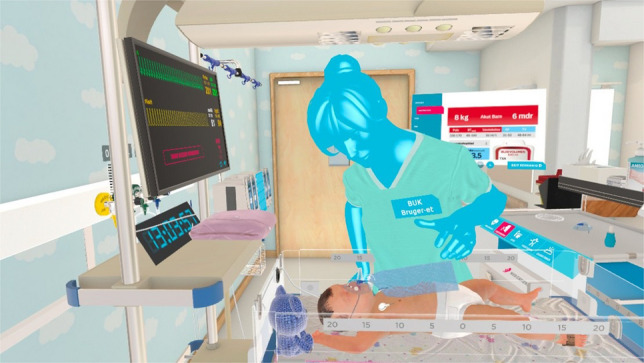
Screenshot from UbiSim© immersive virtual reality paediatric emergency scenario. A trainee doctor and a nurse were immersed in the multiplayer virtual reality simulation, collaborating to stabilise a virtual patient. Each participant was represented as an avatar. Interaction points were integrated into the virtual emergency room, allowing trainees to initiate clinical evaluation and treatments by interacting with virtual equipment. Dropdown menus were available for palpating the patient (e.g. pulse and skin temperature). When interventions were performed, the virtual patient’s condition changed dynamically based on predefined responses in vital signs and clinical presentation. Trainees could move around the room using a teleportation feature

Four immersive VR scenarios were developed, simulating four paediatric emergency cases: sepsis, increased intracranial pressure, respiratory distress, and anaphylaxis. A.M.S., L.P., J.K., and A.P. developed the cases, A.P., J.L.S., L.K.G., and S.L. validated them, and A.M.S. and I.M.H. built them into UbiSim’s © immersive VR platform (Fig. 1) [34]. We used Oculus Quest 2 headsets to deliver the VR scenarios.

#### Development of mannequin-based scenarios for baseline and follow-up assessment

The baseline and follow-up assessment cases depicted an 8-year-old with asthma and a 3-month-old with pneumonia and were designed to be similar in difficulty level. A.M.S., J.K., and A.P. created the cases, with revisions from J.L.S., L.K.G., L.P., and S.L. A.M.S. and J.K. then piloted the cases with trainee doctors, making final adjustments. The dyads were randomly assigned a case at baseline and enacted the alternate case at follow-up.

### Intervention and control

#### Phase 1: Lecture and workshop

Both groups attended an in-person preparatory lecture on ABCDE, acute care communication, introduction to study questionnaires, and a hands-on workshop practising bag-and-mask ventilation and intraosseous access on mannequins (1 h 45 min).

#### Phase 2: Immersive VR training

The *self-guided group* (intervention) completed four interactive, multiplayer VR-based paediatric emergency scenarios. Each scenario was followed by a self-guided debriefing, during which the dyad reflected on the scenario using the PEARLS debriefing script [35], modified based on previous research to fit the VR context [16]. Phase 2 lasted 4 h in total, with each scenario lasting 15–20 min, and 20 min allocated for each debriefing.

The *facilitator-guided group* (control) completed the same four scenarios, but the subsequent debriefings were facilitated by an experienced debriefer, using the same debriefing script as the self-guided group.

### Data collection process

**Fig. 2:**
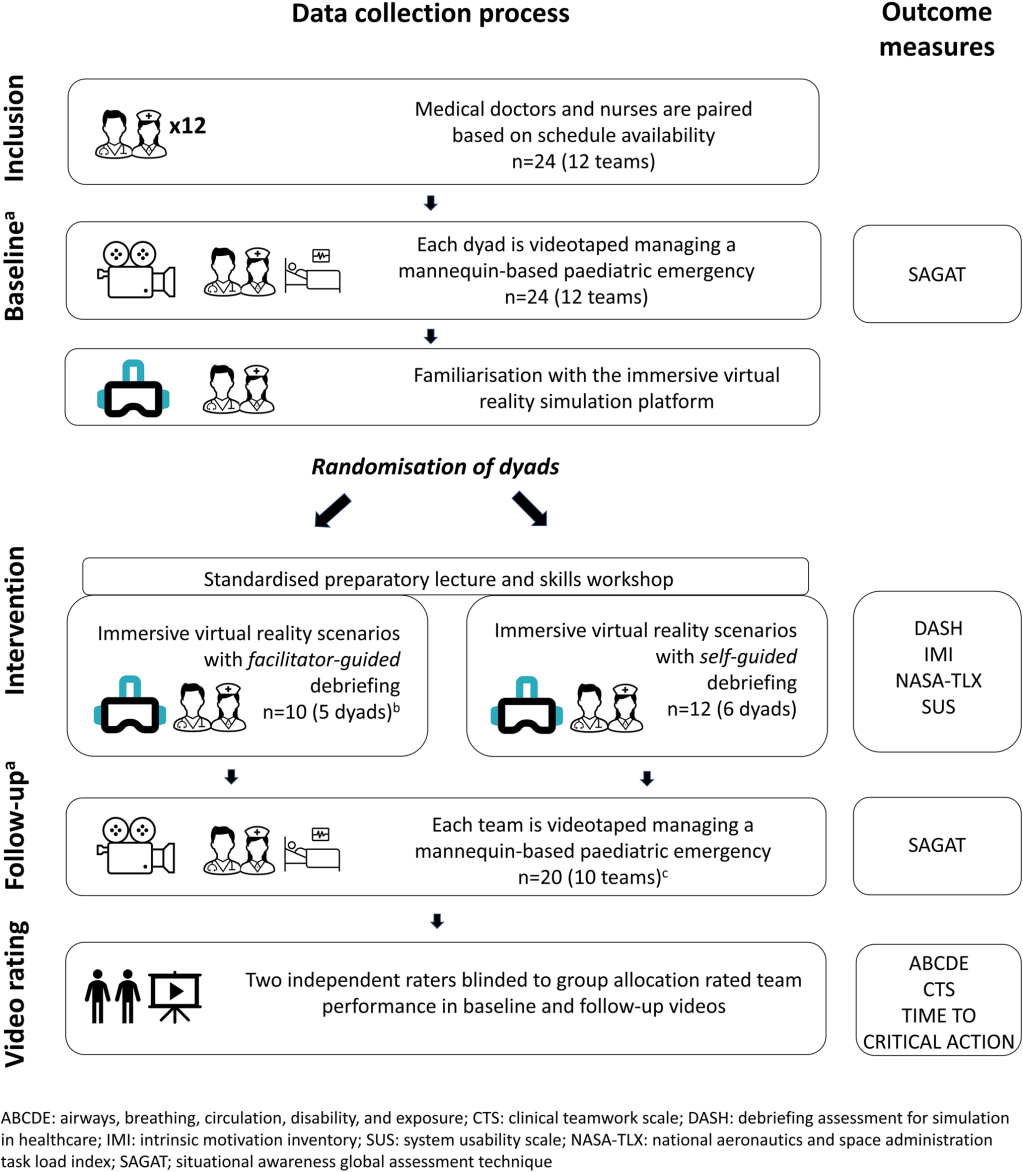
Data collection process. ^a^Baseline was conducted 1 week prior to the intervention. Follow-up was conducted 1 month post-intervention. ^b^One team from the facilitator-guided group dropped out before the intervention day due to short-term sick leave. ^c^One team from the facilitator-guided group dropped out at follow-up due to long-term sick leave

Participant flow, including withdrawals and reasons for withdrawals, is reported in the “Results” section and illustrated in Fig. 2.

The pre- and post-performance assessment scenarios were recorded with a 360 Ricoh THETA Z1 camera and rated independently by two raters (A.M.S. and I.M.H.) blinded to group allocation. Before assessing the videos, the raters reviewed two pilot videos, discussing each subitem and establishing a shared understanding of the tools. Videos were stored on a hospital-based hard drive as a Logged file. All other data were stored in a secure online database (REDCap) [36] and underwent pseudonymisation before being imported into the statistical analysis software (R, version 4.1.2) [37].

### Outcome measures

Baseline data were collected for all participants, including gender, age, clinical experience, and prior experience with simulation, computer games, and immersive VR.

#### Feasibility of the interventions (RQ1a)

Acceptability: measured as participant motivation to engage in VR and debriefing (Intrinsic Motivation Inventory, IMI) and incidence of cybersickness.

Usability: measured using the System Usability Scale (SUS).

Additionally, feasibility issues were recorded by a student observer (technical issues and early termination of scenarios or debriefings).

#### Feasibility of trial procedures for a future randomised controlled trial (RQ1b)

Feasibility indicators included participant recruitment and scheduling of baseline and follow-up visits (number of meetings and email exchanges), the feasibility of video-based assessment of paediatric emergency performance (rating time, rating issues, and interrater reliability), and situational awareness assessment (issues determining the correctness of SAGAT responses).

#### Effect on performance (RQ2)

Assessed by two independent, blinded raters using the Clinical Teamwork Scale ® (CTS) (primary outcome), the ABCDE-checklist, and time to critical actions (secondary outcomes).

#### Perceived effectiveness and workload (RQ3)

Measured using the Debriefing Assessment for Simulation in Healthcare (DASH©) and NASA Task Load Index (NASA-TLX).

The outcome measures are described in detail in Table 1.

**Table 1:** Description of outcome measures

*Feasibility of the interventions (RQ1)*
Cybersickness: We used a single-item question asking the participants to state if they had experienced any cybersickness during or after the immersive VR training and encouraging them to describe the cybersickness
Intrinsic Motivation Inventory (IMI) [38]: This validated questionnaire measures the subjective motivation related to a specific activity on a 7-point Likert scale ranging from not at all true to very true [38]. IMI has previously been translated professionally to Danish and tested in a Danish context [39]
System Usability Scale (SUS) [40]: This validated questionnaire measures the subjective usability of a product on a 5-point Likert-scale ranging from strongly disagree to strongly agree [40].The SUS item scores are subsequently converted into a single SUS score on a scale from 0 to 100 [40]. SUS has previously been translated professionally to Danish and validated in a Danish context
Performance assessment (RQ2)*
ABCDE checklist [41]: Modified from Hultin et al. [41], this checklist assesses adherence to the European Paediatric Advanced Life Support guidelines for ABCDE assessment [42] and ranges from 0 = not initiated to 3 = performed consistently during the whole simulation
Clinical Teamwork Scale (CTS®) [43]: This validated rating scale assesses 15 teamwork in five main domains: overall teamwork, communication, situational awareness, decision-making, and role responsibility, on an 11-point Likert scale ranging from unacceptable to perfect [43]. Two professional translators forward backward translated CTS© to Danish
Situational Awareness Global Assessment Technique (SAGAT) [44, 45]: This validated multiple-choice questionnaire measures learners’ situational awareness during a simulated scenario and the shared situational awareness in a team. Learners are prompted to state the patient’s vitals, primary problem, and appropriate next steps 5 min into the scenario and at the end of the scenario
Time to critical actions: Time to critical actions was measured as time in seconds to administration of oxygen (pneumonia case) or beta2 agonist (asthma case), administration of a fluid bolus (10 mL/kg body weight), and completion of the ABCDE assessment. If thedyad did not perform the action, they were assigned a time of 900 s (maximum length of scenario)
*Perceived effectiveness and workload (RQ3)*
Debriefing Assessment for Simulation in Healthcare Student Version (DASH-SV©) [46]: This validated questionnaire is designed for learners to evaluate a debriefing on a 7-point Likert scale ranging from extremely ineffective to extremely effective [46]. In consultation with the DASH© developers, we replaced ‘The facilitator’ with ‘We’ in the self-guided debriefing group to fit the context. Two professional translators forward backward translated DASH© to Danish
NASA Task Load Index (NASA-TLX) [47]: This validated scale measures subjective workload related to a specific activity. The scale ranges from 0 to 21, and the item scores are subsequently converted into a single score from 0 to 100. Two professional translators forward backward translatedNASA-TLX to Danish

*NASA* National Aeronautics and Space Administration Task Load Index, *RQ* research question

*Two independent raters blinded to group allocation assessed the performance of each interprofessional dyad based on video recordings of the dyad managing a mannequin-based paediatric emergency at baseline and at follow-up

### Statistical analysis

#### Feasibility (RQ1)

IMI and SUS scores were analysed based on a constrained linear mixed model to account for potential correlation between individuals in the same dyad, including allocation as a fixed effect. The number of emails and meetings was presented as descriptive statistics. Interrater reliability between the two raters’ assessments of paediatric emergency performance in the baseline and follow-up assessment cases was determined by the intraclass correlation coefficient.

#### Effect on performance (RQ2)

Pretest–posttest dyad-based outcomes were analysed using a constrained linear mixed model with inherent baseline adjustment. Visit (baseline or follow-up), assessment scenario (pneumonia or asthma), and the constrained interaction between visit and allocation were included as fixed effects. An unstructured covariance pattern was applied to account for the correlation between repeated measurements and potential changes in variance over time. The mean between the two raters was calculated prior to statistical analysis.

#### Perceived effectiveness and workload (RQ3)

DASH and NASA-TLX scores were analysed based on a constrained linear mixed model to account for potential correlation between individuals in the same dyad. Allocation was included as a fixed effect.

Statistical analyses were made using the LMMstar package in the statistical analysis software R [37, 48].

## Results

The study was conducted from September to December 2023. We included a total of 24 participants, 12 trainee doctors, and 12 nurses. Basic characteristics of the intervention and control group were comparable (Table 2). Of the 12 interprofessional doctor–nurse dyads randomised, two dyads in the facilitator-guided debriefing group did not complete the trial. One dyad withdrew prior to the intervention day due to short-term sick leave, and one dyad withdrew before follow-up due to long-term sick leave. Consequently, 11 dyads (22 participants) completed the intervention, and 10 dyads (20 participants) were available for follow-up outcome assessment (Fig. 2).

**Table 2:** Baseline characteristics of participants in the facilitator- and self-guided group (*N* = 24)

	Facilitator-guided	Self-guided
Number of participants	12	12
Number of females/males	11/1	10/2
Median age (range)	28 (24 to 38)	30 (24 to 47)
Number of medical doctors/nurses	6/6	6/6
Median years of clinical work experiences (range)	2.8 (0.17 to 9.0)	3.0 (0.5 to 19.0)
Median years of paediatric work experiences (range)	1.5 (0.17 to 5.0)	1.5 (0.5 to 8.0)
Median simulation experience (range) (1 = no experience, 5 = extensive experience)	3.0 (2.0 to 4.0)	3.5 (1.0 to 4.0)
Median computer game experience (range) (1 = no experience, 5 = extensive experience)	2.0 (1.0 to 3.0)	2.5 (1.0 to 5.0)
Median immersive virtual reality experience (range) (1 = no experience, 5 = extensive experience)	1.0 (1.0 to 3.0)	2.0 (1.0 to 3.0)

### Feasibility of the interventions (RQ1a)

On the first intervention day, five VR headsets failed to log into the scenario module. Affected dyads adapted by having the nurse in VR and the trainee doctor observing and providing supervision via computer screen. Most participants needed brief assistance accessing the correct app and scenario but could independently complete the VR training and debriefing.

Nine of 22 participants (41%) experienced mild, transient cyber sickness, and one (5%) discontinued immersion due to more severe symptoms but continued the training by observing the scenarios on a screen, providing supervision to her teammate.

Mean motivation scores (IMI) were 5.6 (confidence interval (CI) 4.7 to 6.4) in the facilitator-guided group and 5.2 (CI 4.4 to 7.0) in the self-guided group. Mean usability scores were 77.7 (CI 63.6 to 91.8) in the facilitator-based and 73.2 (CI 60. to 86.0) in the self-guided group. Additional data are presented in Table 3, and Online Resource 5 and 7.

**Table 3:** Self-reported outcomes (RQ1 and RQ3)

Allocation group	Mean (CI)^a^	Facilitator versus self-guided group mean difference (CI) and *p*-value^a^
Intrinsic Motivation Inventory (RQ1a). *Scale from 1 to 7*^*b*^		
Facilitator-guided Self-guided	5.6 (4.7 to 6.4) 5.2 (4.4 to 7.0)	0.4 (− 0.8 to 1.6), *p* = 0.47
System Usability Scale (RQ1a). *Score from 0 to 100b*		
Facilitator-guided Self-guided	77.7 (63.6 to 91.8) 73.2 (60. to 86.0)	4.5 (− 14.5 to 23.4), *p* = 0.60
Debriefing Assessment for Simulation in Healthcare (RQ3). *Scale from 1 to 7*^*b*^		
Facilitator-guided Self-guided	6.3 (5.4 to 7.2) 4.9 (4.1 to 5.8)	1.4 (0.1 to 2.7), *p* = 0.04
NASA Task Load Index (RQ3). *Score from 0 to 100*^*b*^		
Facilitator-guided Self-guided	47.3 (34.3 to 60.2) 48.1 (34.7 to 61.4)	− 0.8 (− 18.5 to 16.9), *p* = 0.92

*CI* confidence interval, *NASA* National Aeronautics and Space Administration Task Load Index, *RQ* research question

^a^Based on constrained linear mixed model analysis

^b^Number of participants included in analysis: Facilitator-guided group: *n* = 10; self-guided group: *n* = 12. The mean represents the average score of all subscale items on the scale

### Feasibility of trial procedures for a future randomised controlled trial (RQ1b)

Findings related to RQ1b reflect the feasibility of organising and conducting the trial procedures, including recruitment, scheduling of baseline and follow-up assessments across units, and outcome assessment logistics. Recruiting and coordinating dyads of trainee doctors and nurses from four paediatric units to attend baseline and follow-up visits required collaboration with seven staffing coordinators overseeing work schedules and shift assignments. Scheduling 12 interprofessional dyads (24 participants) involved 12 meetings and 182 emails. Due to last-minute changes in shifts, illness, and department workload, dyads were rescheduled an average of three times (range: 0–6), requiring an average of 27 additional email exchanges per dyad (range: 0–60). These challenges were related to coordinating baseline and follow-up assessments rather than the intervention sessions.

Video quality was sufficient for rating paediatric emergency performance using the CTS©, ABCDE checklist, and time to critical actions. Rating a single 10–15-min video across all three outcomes took an average of 45 min (range 35–57 min). The intraclass correlation coefficient for average measures was 0.97 (CI 0.94 to 0.99) for CTS®, 0.98 (CI 0.97 to 0.99) for the ABCDE checklist, and 0.93 (CI 0.84 to 0.97) for time to critical actions, indicating excellent inter-rater reliability across all three paediatric emergency performance measures.

Due to the dynamic nature of the scenarios, SAGAT responses could not be validated. Constant changes in vital signs and case complexity prevented reliable assessment of correctness of SAGAT responses, and SAGAT data were excluded from analysis.

### Effect of facilitator-guided versus self-guided debriefing on performance (RQ2a)

CTS® (primary outcome): We found no significant difference between the facilitator-guided and self-guided debriefing groups in teamwork, measured as delta CTS®.

ABCDE adherence and time to critical actions: We found no significant difference between the facilitator-guided and self-guided debriefing groups for ABCDE adherence or time to critical actions, measured as delta ABCDE-checklist.

Additional data are presented in Table 4 and Online Resource 1, 2, and 3.

**Table 4:** Effect on performance (RQ2)

Allocation group	Baseline mean (SD)	Facilitator versus self-guided group mean difference in delta baseline to follow-up (CI)^a,b^	Change from baseline to follow-up, mean (CI)^a,c^
Clinical Teamwork Scale mean score (scale from 0 to 10)			
Facilitator-guided Self-guided	5.6 (0.7) 5.8 (1.3)	− 0.2 (− 1.1 to 0.7), *p* = 0.59	1.2 (0.5 to 1.9), *p* = 0.005 1.4 (0.8 to 2.0), *p* < 0.001
ABCDE checklist mean score (checklist from 0 to 3)			
Facilitator-guided Self-guided	2.1 (0.2) 2.1 (0.5)	0.0 (− 0.6 to 0.6), *p* = 0.93	0.5 (0.1 to 1.0), *p* = 0.02 0.5 (0.1 to 0.9), *p* = 0.01
Time to critical actions mean (in seconds)			
Facilitator-guided Self-guided	402 509	45 (− 111 to 202), *p* = 0.50	− 100 (− 212 to 13), *p* = 0.07 − 145 (− 257 to − 33), *p* = 0.02

*CI *confidence interval, *SD* standard deviation

^a^Analysis based on a constrained linear mixed model with inherent baseline adjustment. Number of dyads included in analysis: Facilitator-guided group: baseline *n*** = **6 dyads, follow-up *n*** = **4 dyads; self-guided group: baseline *n*** = **6 dyads, follow-up *n*** = **6 dyads. The mean represents the average score of all subscale items on the scale

^b^The estimated difference between the facilitator- and self-guided group’s mean change from baseline to follow-up

^c^The estimated mean change from baseline to follow-up in the facilitator- and self-guided group, respectively

### Improvement in performance from baseline to follow-up (RQ2b)

We found a significant improvement from baseline to follow-up in teamwork measured with CTS® (facilitator-guided mean 1.2, CI 0.5 to 1.9, *p* = 0.005; self-guided mean 1.4, CI 0.8 to 2.0, *p* < 0.001) and ABCDE adherence (facilitator-guided mean 0.5, CI 0.1 to 1.0, *p* = 0.02; self-guided mean 0.5, CI 0.1 to 0.9, *p* = 0.01). For time to critical actions, we found a significant improvement from baseline to follow-up in the self-guided group (mean − 145, CI − 257 to − 33, *p* = 0.02).

Additional data are presented in Table 4 and Online Resource 1, 2, and 3.

### Perceived effectiveness and workload (RQ3)

We found that the facilitator-guided group perceived the debriefing to be significantly more effective (DASH) compared to the self-guided group (mean difference 1.4, CI 0.1 to 2.7, *p* = 0.04).

We found no significant difference between the facilitator-guided and self-guided debriefing group for perceived workload (NASA-TLX).

Additional data are presented in Table 3 and Online Resource 4 and 6.

## Discussion

### Summary of main findings

We demonstrated the feasibility of interprofessional immersive VR-based paediatric emergency training with both facilitator- and self-guided debriefing in a clinical setting. Whilst VR was associated with high motivation and usability ratings, nearly half of the participants experienced some degree of cybersickness. Trial procedures were feasible in a pilot setting but involved substantial logistical demands that warrant consideration when planning larger-scale studies. Immersive VR training improved healthcare professionals’ teamwork, ABCDE adherence, and time to critical actions, with no significant differences between facilitator-guided and self-guided debriefing. Cognitive load did not differ between groups; however, healthcare professionals perceived facilitator-guided debriefing as more effective.

### Discussion of findings in relation to existing literature

Research question 1 evaluated the interventions’ acceptability and usability, as well as whether the pilot study supported the feasibility of a future, larger randomised trial. We found that delivering immersive VR training was feasible with both debriefing types. The perceived usability of the VR platform was above average usability scores for digital health apps [49], and participant motivation was high in both debriefing groups and comparable to findings from mannequin-based simulation in similar contexts [39]. Nearly half of the participants experienced some degree of cybersickness, which is somewhat higher than rates reported in previous reviews [50]. Known risk factors include headset misalignment, visual latency issues, and prolonged exposure [50, 51].

Occasional headset failures required adaptation of the intervention, with one participant immersed in VR whilst the other observed on a screen and provided supervision. These experiences emphasise the importance of access to technical support, backup headsets, and alternative instructional plans.

Conducting the trial involved extensive communication and repeated rescheduling of participants, largely driven by clinical workload and short-term staff absence. Although manageable in a pilot setting, these demands should be considered in the design of a future larger randomised controlled trial.

Research question 2 investigated whether immersive VR with self-guided debriefing was comparable to facilitator-guided debriefing in improving paediatric emergency performance. We found that immersive VR training improved teamwork, ABCDE adherence, and time to critical actions, with no significant differences between debriefing methods. All participants received a standardised preparatory lecture and skills workshop prior to the VR-based training. This preparatory training may have contributed to the observed performance improvements; however, it is not expected to influence the comparative findings between facilitator- and self-guided debriefing, as both groups received identical preparation.

Previous studies have shown that VR-based paediatric emergency training can improve *individual skills* amongst healthcare professionals and students, including clinical reasoning [13, 52], recognition of clinical signs of severe illness [10], task prioritisation and triage [10–12, 53], recall of the ABCDE approach [14], and adherence to neonatal resuscitation guidelines [15]. Our study extends this body of research by demonstrating the impact of VR-based paediatric emergency training on *interprofessional performance*, specifically in teamwork and ABCDE adherence.

Previous research has found comparable outcomes between facilitator-guided and self-guided debriefing in the context of mannequin-based and screen-based simulations [30, 31]. Our findings add to this literature by indicating comparable results for facilitator- and self-guided debriefing in the context of immersive VR-based paediatric emergency training.

Self-guided approaches may offer advantages such as allowing time for reflection [54], promoting a sense of ownership and commitment to change [26], and motivating self-regulated learning [27]. However, in the absence of a facilitator, learners with less developed self-regulated learning skills may struggle to frame their experiences effectively [28, 55]. Several studies emphasise that effective self-regulated learning requires appropriate scaffolding [26, 28, 56]. In our study, the structured debriefing script likely provided this support, helping learners engage meaningfully in reflection.

Research question 3 addressed whether healthcare professionals perceived differences in effectiveness and cognitive load between self-guided and facilitator-guided debriefing. We found that facilitator-guided debriefing was perceived as more effective, whereas no difference in perceived cognitive load was observed between the two debriefing methods. This aligns with self-regulated learning theory, emphasising that learners with low self-regulated learning skills can feel insecure evaluating their own performance progress [28]. However, the higher perceived effectiveness of facilitator-guided debriefing may, at least in part, reflect participants’ confidence in conducting the debriefing process rather than actual differences in educational effectiveness.

Based on self-regulated learning and cognitive load theory, we had anticipated that cognitive load would be higher in the self-guided group [29]. However, since VR was a new technology for our participants, the cognitive demands of navigating the virtual environment may have overshadowed any differences resulting from the presence or absence of a facilitator.

### Strengths and limitations

This randomised pilot study has several methodological strengths. We used a robust design with randomisation to two comparable interventions and blinded assessment of performance using validated tools. We also demonstrated the feasibility of delivering immersive VR training and debriefing across multiple paediatric units and professions in a real-world clinical setting.

However, several limitations should be noted. Although feasible in a pilot context, the trial required substantial logistical coordination, and volunteer participation may have introduced selection bias, potentially inflating feasibility and acceptability compared with a less motivated population.

Because the trial was based on a convenience sample, it was not powered to formally test non-inferiority between debriefing methods, limiting conclusions from between-group comparisons.

Another limitation was occasional technical issues that required adaptation of the intervention, which may have influenced cognitive load, motivation, and interprofessional interaction.

The lower self-reported ratings of self-guided debriefing should be interpreted cautiously, as they may reflect participants’ confidence in conducting the debriefing rather than differences in learning outcomes.

Finally, this was a single-centre study conducted at a high-resource hospital. The feasibility and effects of VR and self-guided debriefing may differ in other contexts, particularly those with limited access to equipment, technical support, or prior simulation experience.

### Implications

Logistical challenges related to recruiting and scheduling dyads across multiple units and timepoints highlight the potential value of fewer data collection points or the inclusion of participant groups with greater scheduling flexibility in future studies.

Consistent with previous research, our findings emphasise the importance of backup plans in case of cyber sickness or technical issues, access to technical support, and participant familiarisation when implementing immersive VR training [16, 57–59].

The potential for effective self-guided VR training may ease implementation and reduce the cost of regular simulation-based training. Future trials could compare the two debriefing methods using a non-inferiority design and explore for whom and under what conditions each approach is most effective.

## Conclusion

This randomised pilot study demonstrates that immersive VR-based paediatric emergency training with both facilitator-guided and self-guided debriefing is feasible in a clinical setting. Whilst trial procedures were feasible in a pilot context, logistical demands may pose challenges for larger studies. Self-guided debriefing was associated with improvements in paediatric emergency performance comparable to those achieved with facilitator-guided debriefing, suggesting potential as a resource-efficient debriefing approach. Future adequately powered trials are needed to formally evaluate non-inferiority and to further examine feasibility and implementation across diverse settings.

## Supplementary information

Below is the link to the electronic supplementary material.

ESM 1 (DOCX 125 KB)

## Data Availability

The data that support the findings of this study are  are available from the corresponding author upon reasonable request.

## Abbreviations

ABCDE: Airways, breathing, circulation, disability, and exposure
CI: Confidence intervals
CTS: Clinical teamwork scale
DASH: Debriefing assessment for simulation in healthcare
IMI: Intrinsic Motivation Inventory
REDCap: Research electronic data capture
SUS: System usability scale
NASA-TLX: National aeronautics and space administration task load index
RQ: Research question
SAGAT: Situational awareness global assessment technique
VR: Virtual reality
