# Supplementary material for: Facilitator-guided vs self-guided debriefing in immersive virtual reality paediatric emergency training: a randomised pilot study on learning outcomes and feasibility
Source: Eur J Pediatr. 2026 Apr 2;185(5):234. doi: 10.1007/s00431-026-06898-3 (PMC13046623; doi:10.1007/s00431-026-06898-3)
Supplement: Supplementary file 1 — (DOCX 125 KB) [file 431_2026_6898_MOESM1_ESM.docx]

**SUPPLEMENTARY MATERIAL**

| Supplementary 1: ABCDE checklist descriptive statistics for each subscale item | | | | | | |
| --- | --- | --- | --- | --- | --- | --- |
|  | Checklist from 0 = not initiated to 3 = performed consistently during the whole simulation | | | | | |
| ABCDE subscale item | **Allocation group** | **Baseline** | | **Follow-up** | |  |
|  |  | **Mean (SD)** | **Median (IQR)** | **Mean (SD)** | **Median (IQR)** |  |
| AIRWAY assessment | | | | | | |
| Airway assessment | **Facilitator-guided Self-guided** | 2.8 (0.4) 2.7 (0.8) | 3.0 (0.0) 3.0 (0.0) | 3.0 (0.0) 2.7 (0.5) | 3.0 (0.0) 3.0 (0.8) |  |
| establish patent airway | **Facilitator-guided Self-guided** | 2.3 (0.6) 2.9 (0.3) | 3.0 (0.5) 3.0 (0.1) | 3.0 (0.0) 2.5 (0.7) | 3.0 (0.0) 2.8 (0.5) |  |
| BREATHING assessment | | | | | | |
| Oxygenation | **Facilitator-guided Self-guided** | 2.8 (0.4) 3.0 (0.0) | 3.0 (0.0) 3.0 (0.0) | 3.0 (0.0) 3.0 (0.0) | 3.0 (0.0) 3.0 (0.0) |  |
| Respiratory rate | **Facilitator-guided Self-guided** | 2.2 (1.3) 1.5 (1.4) | 3.0 (1.1) 1.5 (2.6) | 3.0 (0.0) 2.2 (0.9) | 3.0 (0.0) 2.5 (1.4) |  |
| Tidal volume | **Facilitator-guided Self-guided** | 3.0 (0.0) 2.9 (0.2) | 3.0 (0.0) 3.0 (0.0) | 3.0 (0.0) 3.0 (0.0) | 3.0 (0.0) 3.0 (0.0) |  |
| Work of breathing | **Facilitator-guided Self-guided** | 2.7 (0.8) 2.5 (0.8) | 3.0 (0.0) 3.0 (1.1) | 2.5 (0.6) 2.4 (1.2) | 2.5 (1.0) 3.0 (0.4) |  |
| Relevant treatment interventions | **Facilitator-guided Self-guided** | 2.7 (0.5) 2.6 (0.5) | 3.0 (0.8) 2.8 (0.9) | 2.8 (0.5) 3.0 (0.0) | 3.0 (0.2) 3.0 (0.0) |  |
| circulation assessment | | | | | | |
| Blood pressure | **Facilitator-guided Self-guided** | 2.0 (1.2) 2.6 (0.4) | 2.5 (1.5) 2.5 (0.4) | 2.8 (0.5) 2.9 (0.2) | 3.0 (0.2) 3.0 (0.0) |  |
| Pulse rate | **Facilitator-guided Self-guided** | 2.6 (0.4) 2.9 (0.2) | 2.5 (0.4) 3.0 (0.0) | 2.8 (0.5) 2.8 (0.3) | 3.0 (0.2) 3.0 (0.4) |  |
| Pulse volume | **Facilitator-guided Self-guided** | 1.2 (1.3) 1.0 (1.5) | 1.0 (2.0) 1.0 (2.2) | 2.2 (1.5) 2.4 (1.2) | 3.0 (0.8) 3.0 (0.4) |  |
| Perfusion | **Facilitator-guided Self-guided** | 3.0 (0.0) 2.7 (0.8) | 3.0 (0.0) 3.0 (0.0) | 3.0 (0.0) 2.9 (0.2) | 3.0 (0.0) 3.0 (0.0) |  |
| Preload | **Facilitator-guided Self-guided** | 1.8 (1.4) 2.3 (1.2) | 2.2 (2.4) 2.8 (0.5) | 2.8 (0.5) 2.7 (0.6) | 3.0 (0.2) 3.0 (0.4) |  |
| Relevant interventions | **Facilitator-guided Self-guided** | 2.0 (0.8) 1.5 (1.0) | 2.2 (1.3) 1.5 (1.4) | 2.5 (0.6) 1.9 (0.4) | 2.5 (1.0) 2.0 (0.0) |  |
| Disability assessment | | | | | | |
| Blood glucose | **Facilitator-guided Self-guided** | 1.8 (1.1) 1.2 (1.2) | 2.0 (1.4) 1.0 (1.6) | 2.4 (0.3) 1.9 (0.6) | 2.5 (0.1) 2.0 (0.8) |  |
| Pupils | **Facilitator-guided Self-guided** | 0.9 (1.4) 1.0 (1.5) | 0.0 (1.9) 0.0 (2.2) | 2.2 (1.5) 1.8 (1.4) | 3.0 (0.8) 2.5 (2.3) |  |
| consciousness Level | **Facilitator-guided Self-guided** | 2.3 (0.4) 2.8 (0.4) | 2.2 (0.5) 3.0 (0.4) | 2.9 (0.3) 2.8 (0.4) | 3.0 (0.1) 3.0 (0.0) |  |
| Posturing or focal signs | **Facilitator-guided Self-guided** | 0.0 (0.0) 0.6 (1.2) | 0.0 (0.0) 0.0 (0.4) | 0.0 (0.0) 1.0 (1.5) | 0.0 (0.0) 0.0 (2.2) |  |
| Exposure assessment | | | | | | |
| Expose skin | **Facilitator-guided Self-guided** | 1.0 (1.5) 1.0 (1.5) | 0.0 (2.2) 0.0 (2.2) | 1.5 (1.7) 2.0 (1.5) | 1.5 (3.0) 3.0 (2.2) |  |
| Temperature | **Facilitator-guided Self-guided** | 2.2 (1.3) 1.9 (1.5) | 3.0 (1.5) 2.8 (2.4) | 2.4 (1.2) 3.0 (0.0) | 3.0 (0.6)  3.0 (0.0) |  |
| ABCDE GLOBAL SCORE (1-7) | | | | | | |
|  | **Facilitator-guided Self-guided** | 4.0 (0.45) 4.2 (1.5) | 4,0 (0.8) 3.8 (2.6) | 5,8 (1.26) 5.7 (1.5) | 6 (0.7) 6.0 (1.6) |  |
| IQR: interquartile range | | | | | | |

| Supplementary 2: Clinical Teamwork Scale Descriptive statistics for each subscale item | | | | | |
| --- | --- | --- | --- | --- | --- |
| Scale from 0 = unacceptable to 10 = perfect | | | | | |
| CTS subscale items | **Allocation group** | **Baseline** | | **Follow-up** | |
|  |  | **Mean (SD)** | **Median (IQR)** | **Mean (SD)** | **Median (IQR)** |
| Overall | | | | | |
| Overall teamwork | **Facilitator-guided Self-guided** | 5.7 (1.1) 6.0 (1.8) | 5.8 (1.3) 6.2 (7.4) | 7.0 7.5 | 7 (0.4) 8 (1.5) |
| Communication | | | | | |
| Overall communication | **Facilitator-guided Self-guided** | 5.5 (1.2) 5.3 (1.4) | 5.5 (1.8) 5.2 (1.5) | 6.8 (1.0) 7.2 (1.5) | 6.5 (1.2) 7.5 (2.2) |
| Orient new members | **Facilitator-guided Self-guided** | NA NA | NA NA | NA NA | NA NA |
| Transparent thinking | **Facilitator-guided Self-guided** | 6.2 (1.1) 6.2 (1.9) | 6.5 (1.1) 6.0 (2.6) | 8.0 (0.8) 7.8 (1.6) | 8.0 (0.4) 8.2 (1.7) |
| Directed communication | **Facilitator-guided Self-guided** | 5.1 (1.4) 4.8 (1.0) | 5.5 (1.8) 4.8 (1.5) | 5.0 (1.5) 6.6 (1.2) | 5.2 (1.3) 6.2 (2.0) |
| Closed loop communication | **Facilitator-guided Self-guided** | 5.8 (1.3) 4.8 (1.4) | 6.2 (1.3) 4.5 (1.5) | 6.1 (2.2) 6.3 (2.1) | 6.0 (3.4) 6.8 (3.2) |
| Situational Awareness | | | | | |
| Overall Situational Awareness | **Facilitator-guided Self-guided** | 5.8 (1.2) 6.6 (2.3) | 5.5 (1.5) 7.8 (3.5) | 7.5 (1.3) 7.5 (1.5) | 7.5 (1.4) 8.0 (1.7) |
| Resource allocation | **Facilitator-guided Self-guided** | 5.2 (1.4) 6.7 (2.2) | 5.5 (1.8) 7.5 (3.3) | 7.4 (1.0) 8.0 (0.8) | 7.8 (0.9) 8.2 (1.3) |
| Decision Making | | | | | |
| Overall Decision Making Rating: | **Facilitator-guided Self-guided** | 5.8 (1.3) 6.0 (1.9) | 5.8 (1.5) 6.5 (3.1) | 7.0 (1.4) 7.8 (1.7) | 7.5 (1.5) 8.5 (2.1) |
| Prioritize | **Facilitator-guided Self-guided** | 5.0 (1.4) 5.8 (2.3) | 5.0 (2.1) 6.8 (3.7) | 7.0 (1.2) 7.2 (1.9) | 7.0 (2.0) 7.8 (2.0) |
| Role Responsibility | | | | | |
| Overall Role Responsibility | **Facilitator-guided Self-guided** | 6.4 (0.7) 6.8 (1.5) | 6.2 (0.9) 7.5 (1.9) | 7.6 (1.0) 7.5 (1.5) | 7.2 (0.9) 8.2 (1.6) |
| Role clarity | **Facilitator-guided Self-guided** | 5.3 (0.6) 5.5 (0.6) | 5.5 (1.4) 5.8 (0.9) | 6.0 (0.0) 6.5 (1.0) | 6.0 (6.0) 6.0 (1.1) |
| Perform as a leader | **Facilitator-guided Self-guided** | 5.2 (1.2) 6.3 (2.0) | 5.0 (0.8) 7.0 (3.1) | 7.4 (0.5) 7.5 (1.5) | 7.2 (0.6) 8.2 (1.6) |
| Perform as a helper | **Facilitator-guided Self-guided** | 6.6 (1.0) 6.5 (1.8) | 6.8 (0.9) 6.8 (2.0) | 7.8 (1.7) 7.8 (1.1) | 8.0 (1.0) 7.2 (1.6) |
| Other | | | | | |
| Patient friendly | **Facilitator-guided Self-guided** | 4.0 (1.7) 4.2 (1.5) | 3.8 (1.3) 4.0 (1.4) | 4.5 (1.1) 6.1 (0.9) | 4.2 (1.0) 6.0 (0.8) |
| IQR: interquartile range, CTS: Clinical teamwork scale | | | | | |

| \| Supplementary 3: time to critical action descriptive statistics* \| \| \| \| \| \| \| \| --- \| --- \| --- \| --- \| --- \| --- \| --- \| \| Critical action* \| **Allocation group** \| **Baseline** \| \| **Follow-up** \| \|  \| \|  \|  \| **Mean (SD)** \| **Median (IQR)** \| **Mean (SD)** \| **Median  (IQR)** \|  \| \| Time to abcde \| **Facilitator-guided Self-guided** \| 502 (254) 537 (319) \| 444 (312) 504 (577) \| 414 (189) 396 (130) \| 384 (214) 394 (160) \|  \| \| time to oxygen (pneumonia case) \| **Facilitator-guided Self-guided** \| 89 (31) 379 (357) \| 72 (27) 244 (307) \| 302 (222) 123 (41) \| 302 (157)  123 (29) \|  \| \| time to beta 2 agonist (asthma case) \| **Facilitator-guided Self-guided** \| 227 (88) 179 (7) \| 202 (86) 179 (5) \| 143 (74) 177 (18) \| 169 (52) 178 (28) \|  \| \| Time to fluid bolus 10ml/kg \| **Facilitator-guided Self-guided** \| 545 (210) 678 (250) \| 498 (173) 728 (432) \| 376 (129) 488 (210) \| 370 (202) 428 (68) \|  \| \| *Time is presented in seconds IQR: interquartile range \| \| \| \| \| \|  \|  \| Supplementary 4: Debriefing Assessment for Simulation in Healthcare*  (1= extremely ineffective, 7=extremely effective) \| \| \| \| \| --- \| --- \| --- \| --- \| \|  \|  \| **Mean (SD)** \| **Median (IQR)** \| \| The facilitator/we established an engaging learning experience \| Facilitator-guided Self-guided \| 6.0 (0.6) 4.9 (1.4) \| 6.0 (1.0) 4.8 (1.8) \| \| The facilitator/we maintained an engaging context for learning \| Facilitator-guided Self-guided \| 6.4 (0.5) 5.1 (1.1) \| 6.4 (0.7) 4.9 (1.6) \| \| The facilitator/we structured the debriefing in an organized way \| Facilitator-guided Self-guided \| 6.4 (0.5) 5.0 (1.0) \| 6.4 (0.7) 4.8 (1.0) \| \| The facilitator/we provoked in-depth discussions that made me reflect on my performance. \| Facilitator-guided Self-guided \| 6.5 (0.4) 4.8 (1.1) \| 6.7 (0.7) 4.5 (1.1) \| \| The facilitator/we identified what I did well or poorly – and why. \| Facilitator-guided Self-guided \| 6.3 (0.6) 4.8 (1.2) \| 6.4 (1.1) 4.8 (1.3) \| \| The facilitator/we helped me see how I could improve or maintain a good performance \|  \| 6.5 (0.4) 4.9 (1.2) \| 6.4 (0.8) 4.7 (5.2) \| \| *For the Self-guided group, 'The facilitator' was replaced with 'We' to fit the context. The modification was made in consultation with the Debriefing Assessment for Simulation in Healthcare© developers. IQR: interquartile range \| \| \| \|  \| Supplementary 5: iNTRINSIC MOTIVATION INVENTORY DESCRIPTIVE STATISTICS FOR EACH DOMAIN (1= NOT AT ALL TRUE, 7=VERY TRUE) \| \| \| \| \| --- \| --- \| --- \| --- \| \| **Subscale category** \| **Allocation group** \| **Mean (SD)** \| **Median (IQR)** \| \| Interest/enjoyment \| Facilitator-guided Self-guided \| 6.2 (0.8) 5.2 (1.1) \| 6.4 (0.9) 4.9 (1.5) \| \| Perceived competence \| Facilitator-guided Self-guided \| 4.5 (0.8) 4.2 (1.2) \| 4.8 (1.0) 4.2 (2.6) \| \| Perceived choice \| Facilitator-guided Self-guided \| 5.8 (1.0) 5.6 (1.2) \| 6.2 (1.5) 6.1 (1.9) \| \| Pressure tension \| Facilitator-guided Self-guided \| 3.2 (1.3) 3.0 (1.3) \| 2.8 (1.7) 2.8 (1.8) \| \| IQR: interquartile range \| \| \| \| \|  \| \| \| \|  \| SUPPLEMENTARY 6: NASA TASK LOAD INDEX DESCRIPTIVE STATISTICS FOR EACH SUBSCALE ITEM \| \| \| \| \| --- \| --- \| --- \| --- \| \| (Scores from 0-100 from low to high*) \| \| \| \| \|  \|  \| **Mean (SD)** \| **Median (IQR)** \| \| Mental demand \| Facilitator-guided Self-guided \| 70 (20) 59 (24) \| 75 (21) 62 (15) \| \| Physical demand \| Facilitator-guided Self-guided \| 15 (16) 23 (23) \| 15 (24) 20 (17) \| \| Temporal demand \| Facilitator-guided Self-guided \| 52 (11) 49 (12) \| 50 (4) 50 (1) \| \| Performance \| Facilitator-guided Self-guided \| 44 (26) 35 (26) \| 42 (37) 22 (32) \| \| Effort \| Facilitator-guided Self-guided \| 68 (18) 66 (19) \| 68 (10) 65 (17) \| \| Frustration \| Facilitator-guided Self-guided \| 28 (31) 39 (29) \| 22 (42) 25 (34) \| \| * The scale comprises 21 vertical tick marks for each subscale item, each of which represent an interval of 5, such that the scale goes from 0-100 IQR: interquartile range \| \| \| \| |
| --- | --- | --- | --- | --- | --- | --- | --- | --- | --- | --- | --- | --- | --- | --- | --- | --- | --- | --- | --- | --- | --- | --- | --- | --- | --- | --- | --- | --- | --- | --- | --- | --- | --- | --- | --- | --- | --- | --- | --- | --- | --- | --- | --- | --- | --- | --- | --- | --- | --- | --- | --- | --- | --- | --- | --- | --- | --- | --- | --- | --- | --- | --- | --- | --- | --- | --- | --- | --- | --- | --- | --- | --- | --- | --- | --- | --- | --- | --- | --- | --- | --- | --- | --- | --- | --- | --- | --- | --- | --- | --- | --- | --- | --- | --- | --- | --- | --- | --- | --- | --- | --- | --- | --- | --- | --- | --- | --- | --- | --- | --- | --- | --- | --- | --- | --- | --- | --- | --- | --- | --- | --- | --- | --- | --- | --- | --- | --- | --- | --- | --- | --- | --- | --- | --- | --- | --- | --- | --- | --- | --- | --- | --- | --- | --- | --- | --- | --- | --- | --- | --- | --- | --- | --- | --- | --- | --- | --- | --- | --- | --- | --- | --- | --- | --- |

| SUPPLEMENTARY 7: SYSTEM USABILITY SCALE DESCRIPTIVE STATISTICS FOR EACH SUBSCALE ITEM (1=strongly disagree, 5=strongly agree) | | | |
| --- | --- | --- | --- |
|  |  | **Mean (SD)** | **Median (IQR)** |
| I think that I would like to use this VR simulation frequently | Facilitator-guided Self-guided | 4.7 (0.7) 3.5 (1.1) | 5.0 (0.0) 3.5 (1.0) |
| I thought the VR simulation was easy to use | Facilitator-guided Self-guided | 3.9 (0.6) 3.8 (1.0) | 4.0 (1.0) 4.0 (1.2) |
| I found the various functions in the VR were well-integrated | Facilitator-guided Self-guided | 4.0 (0.5) 3.6 (1.1) | 4.0 (0.0) 4.0 (1.0) |
| I imagine that most people would learn to use this VR simulation very quickly | Facilitator-guided Self-guided | 3.8 (1.0) 4.0 (1.1) | 4 (0.8) 4 (1.0) |
| I felt very confident using the VR | Facilitator-guided Self-guided | 3.4 (1.0) 3.9 (1.0) | 4 (1.0) 4 (2.0) |
| **Reverse coded items** | | | |
| I found the VR simulation unnecessarily complex | Facilitator-guided Self-guided | 1.7 (0.7) 1.8 (1.0) | 2.0 (1.0)  1.5 (1.2) |
| I think that I would need the support of a technical person to be able to use this product | Facilitator-guided Self-guided | 2.5 (0.9) 2.5 (0.3) | 3 (0.8) 2 (1.0) |
| I thought there was too much inconsistency in the VR simulation | Facilitator-guided Self-guided | 1.4 (0.5) 2.0 (1.0) | 2.0 (1.0) 2.2 (1.2) |
| I found the VR very awkward to use | Facilitator-guided Self-guided | 1.7 (0.8) 1.8 (1.0) | 1.5 (1.0) 1.0 (1.2) |
| I needed to learn a lot of things before I could get going with the VR | Facilitator-guided Self-guided | 2.2 (1.1) 1.8 (1.1) | 2.0 (2.0) 1.5 (1.0) |
| **Overall, I would rate the user-friendliness of this product as:**  **1 worst imaginable, 2 awful, 3 poor, 4 ok, 5 good, 6 excellent, 7 best imaginable** | | | |
|  | Facilitator-guided Self-guided | 5.5 (0.7) 5.2 (1.0) | 6 (1.0) 5 (1.0) |

IQR: interquartile range
